# Vitamin D in liquid food supplements: are labels in line with RDA?

**DOI:** 10.1080/07853890.2021.1896145

**Published:** 2021-09-28

**Authors:** R. Inez, A. Figueiredo, I. M. Costa, M. D. Auxtero

**Affiliations:** a PharmSci Lab, Centro de Investigação Interdisciplinar Egas Moniz (CiiEM), Egas Moniz Cooperativa de Ensino Superior, Caparica, Portugal; b Centro de Investigação Interdisciplinar Egas Moniz (CiiEM), Egas Moniz Cooperativa de Ensino Superior, Caparica, Portugal

## Abstract

**Introduction:**

Nowadays, it has been observed an increase consumption in vitamins and food supplements (FS). In Portugal, in 2018, more than 2 million individuals reported the intake of these products [1]. Media has been given a particular attention to the high prevalence of vitamin D (VitD) deficiency, which may explain its highest consumption [2]. This vitamin increases intestinal calcium absorption and plays a central role in its homeostasis. Although vitD toxicity is uncommon, being a fat-soluble vitamin, excessive supplementation may result in body accumulation and toxicity [3]. The aim of this study is to evaluate if daily dose of vitamin D claimed in FS labels is in conformity with the recommended daily allowances (RDA) for this vitamin defined by European Union Directive and Portuguese legislation [4].

**Materials and methods:**

A total of 65 FS sold in Portuguese pharmacies, health shops, supermarkets and on the internet were examined for indicated daily intake and dosage of vitamin D. Selection criteria included: oral liquid pharmaceutical forms, for adults or paediatric consumption containing vitD in its composition, as mentioned in the label, regardless of the purpose of the FS.

**Results:**

35 (54%) FS presented vitD label doses above RDA and six (9%) of them indicated a daily dose ≥ the tolerable upper intake level defined by EFSA (UL = 100 μg/day) (Figure 1).

**Discussion and conclusions:**

VitD label dose far exceeded RDA value in most of the FS evaluated and some exceeded UL defined by EFSA. Currently, the economic operators who place FS on the market are the responsible for the safety and the authenticity of label data. Attending that these products are often taken without any medical supervision or counselling and vitD excess may trigger adverse effects and also considering that some of these liquid formulations are for children consumption, it increases the concern about FS safety, it is imperative that the daily doses of this vitamin are reviewed in FS, in accordance to RDA values. FS should be under the same quality control of pharmaceuticals, regarding FS consumers health.

**Figure 1.**
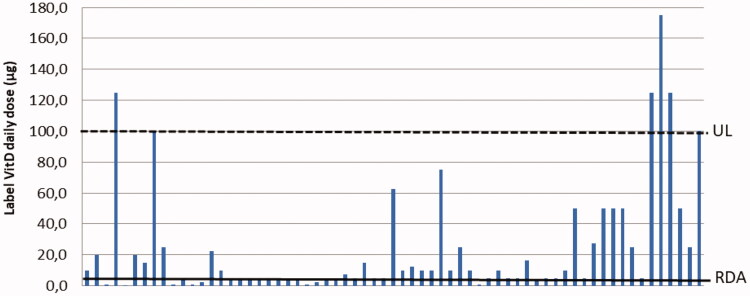
NIR calibration curve used to predict the amorphous fraction of OLZ.
